# Supplementary material for: A Comparison of the Photophysical, Electrochemical and Cytotoxic Properties of meso-(2-, 3- and 4-Pyridyl)-BODIPYs and Their Derivatives
Source: Sensors (Basel). 2022 Jul 7;22(14):5121. doi: 10.3390/s22145121 (PMC9315496; doi:10.3390/s22145121)
Supplement: Supplementary file 1 [file sensors-22-05121-s001.zip › sensors-1753344-supplementary.pdf]

Supplementary Materials

A Comparison of the Photophysical and Electrochemical Properties of *meso*-(2-, 3- and 4-Pyridyl)-BODIPYs and Their Derivatives

Caroline Ndung'U [1], Daniel J. LaMaster [1], Simran Dhingra [1], Nathan H. Mitchell [1], Petia Bobadova-Parvanova, [2] Frank R. Fronczek [1], Noémie Elgrishi [1] and Maria da Graça H. Vicente [1,*]

[1] Department of Chemistry, Louisiana State University, Baton Rouge, LA 70803, USA
[2] Department of Chemistry and Fermentation Sciences, Appalachian State University, Boone, NC 28608, USA
* Correspondence: vicente@lsu.edu

Table of Contents

Spectroscopic data in methanol 2
Absorption and emission spectra 3
Dark and phototoxicity 3
^{1}H, ^{13}C and ^{11}B NMR spectra 4

Table S1. Relative fluorescence quantum yields determined using rhodamine-6G (Φ=0.86) in methanol, λ_{exc}=473nm

Solvent	BODIPY	λ_{abs} (nm)	λ_{em} (nm)	Stokes Shift (nm)	Φ_f	ε (M^{-1} cm $^{-1}$)
CH_3OH	**2Py**	504	514	10	0.13	119800
	**3Py**	503	515	12	0.34	98140
	**$2PyCl_2$**	531	548	17	0.23	69940
	**$3PyCl_2$**	530	546	16	0.57	66880

(a)

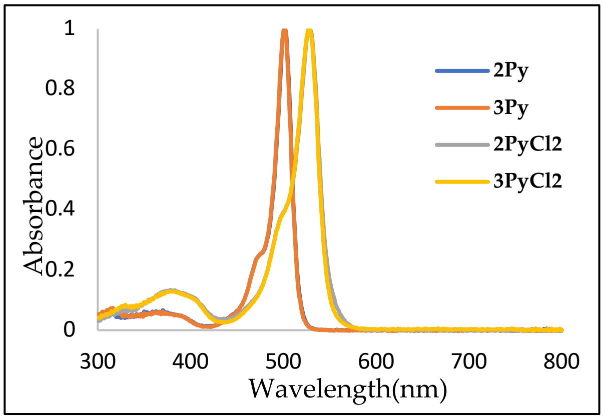

(b)

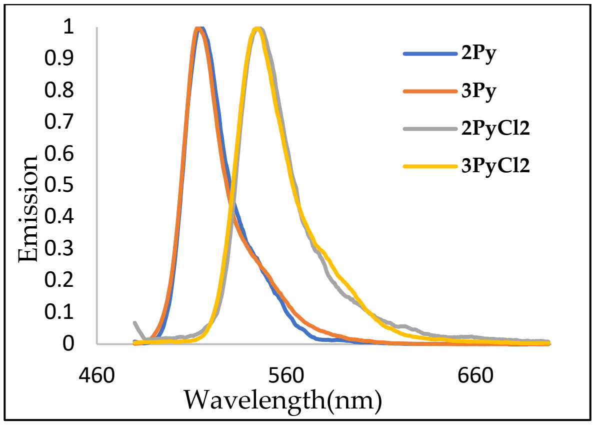

(c)

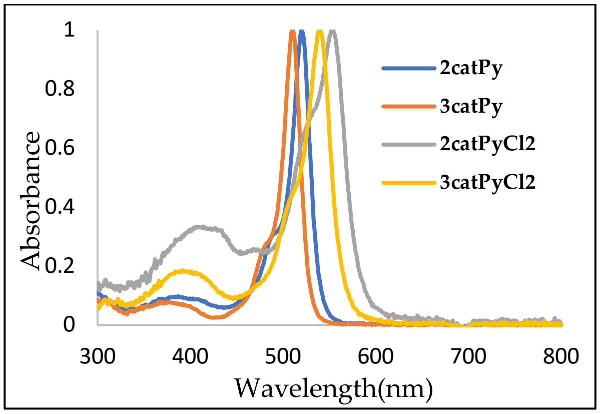

(d)

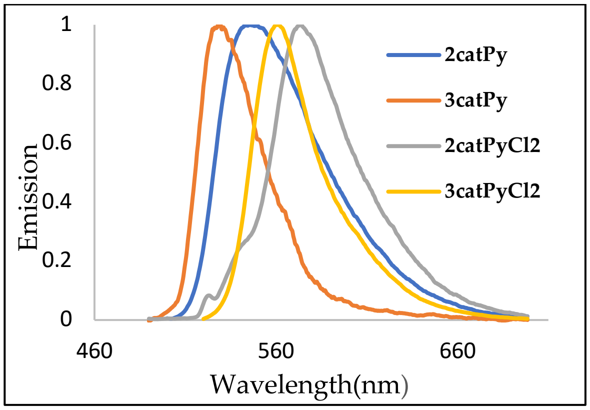

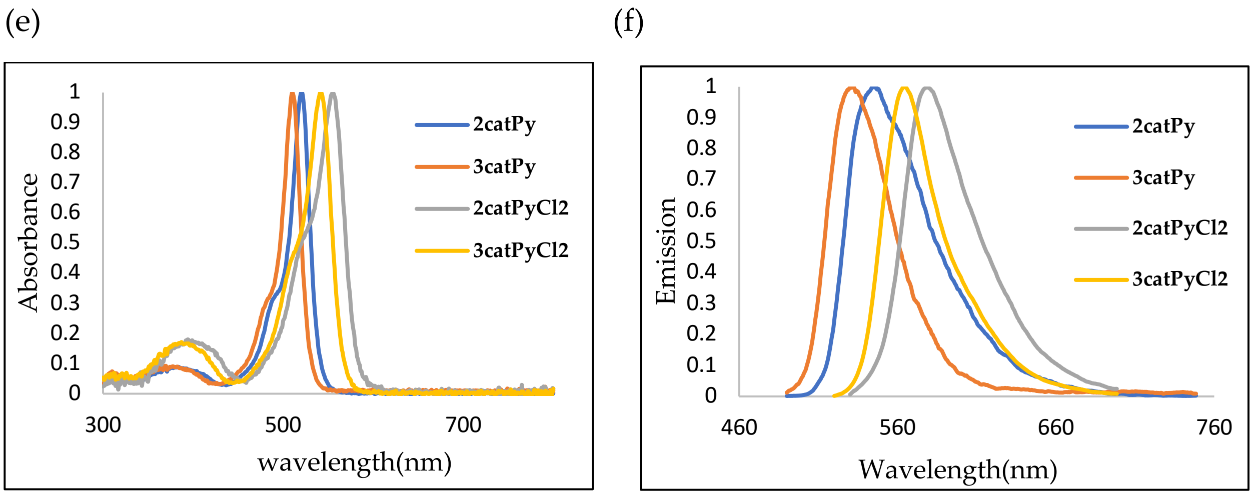

Figure S1. Normalized absorption (a, c, e) and emission (b, d, f) spectra of BODIPYs in acetonitrile (a, b, c, d) and water (e, f).

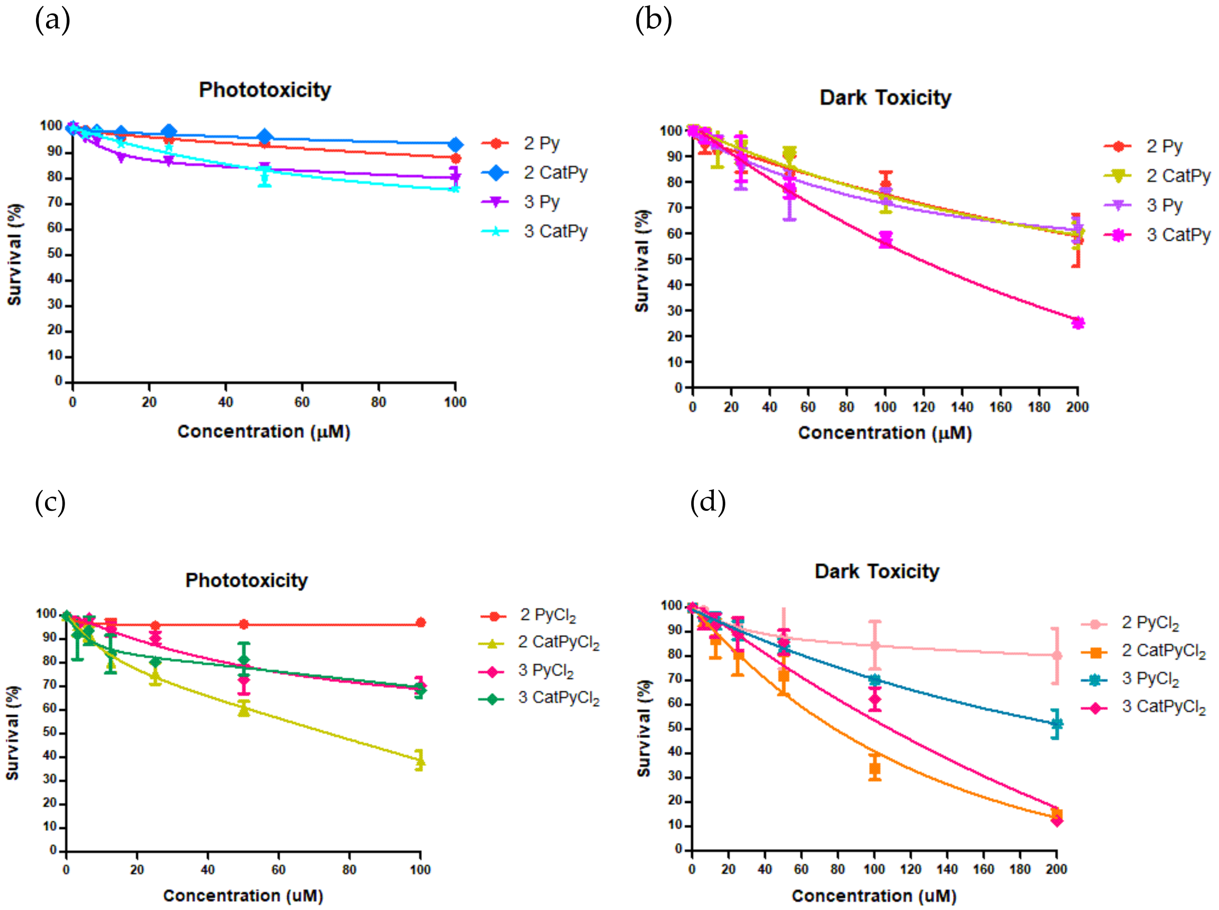

Figure S2. Dark (b, d) and photo (a, c) (1.5 J/cm^2) cytotoxicity of BODIPYs towards HEp2 cells.

NMR

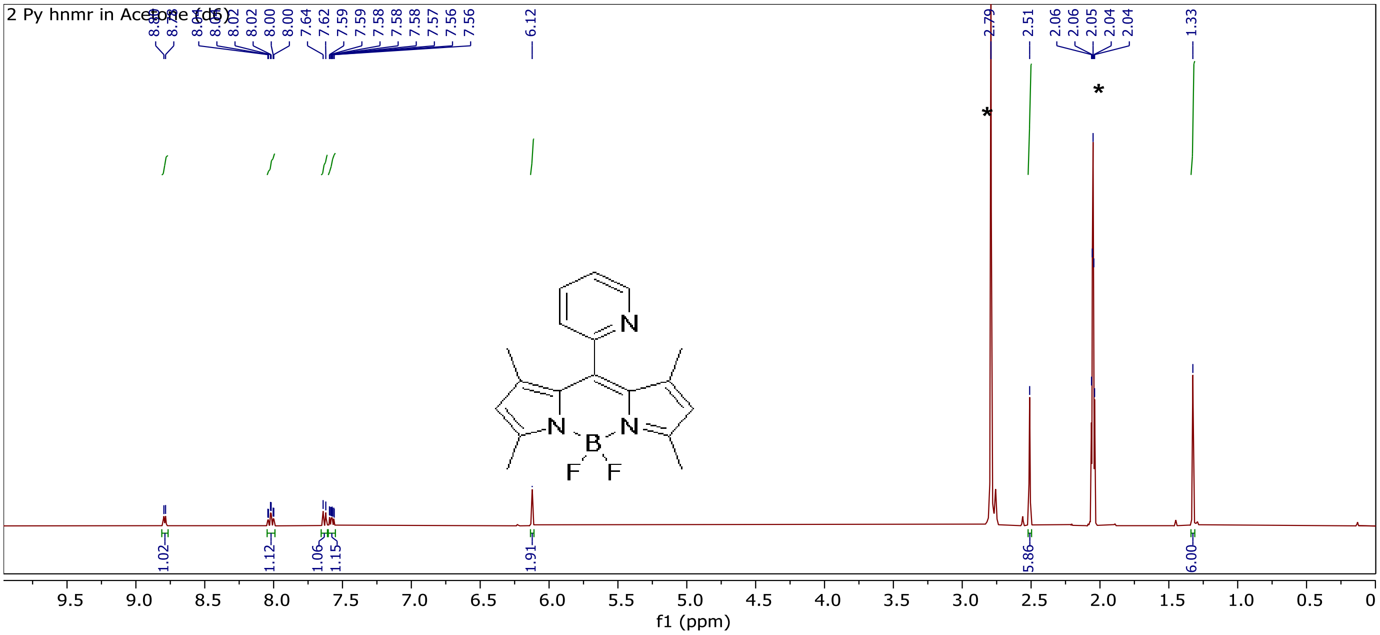

Figure S3. ^{1}H NMR of BODIPY **2Py** in acetone-d_6

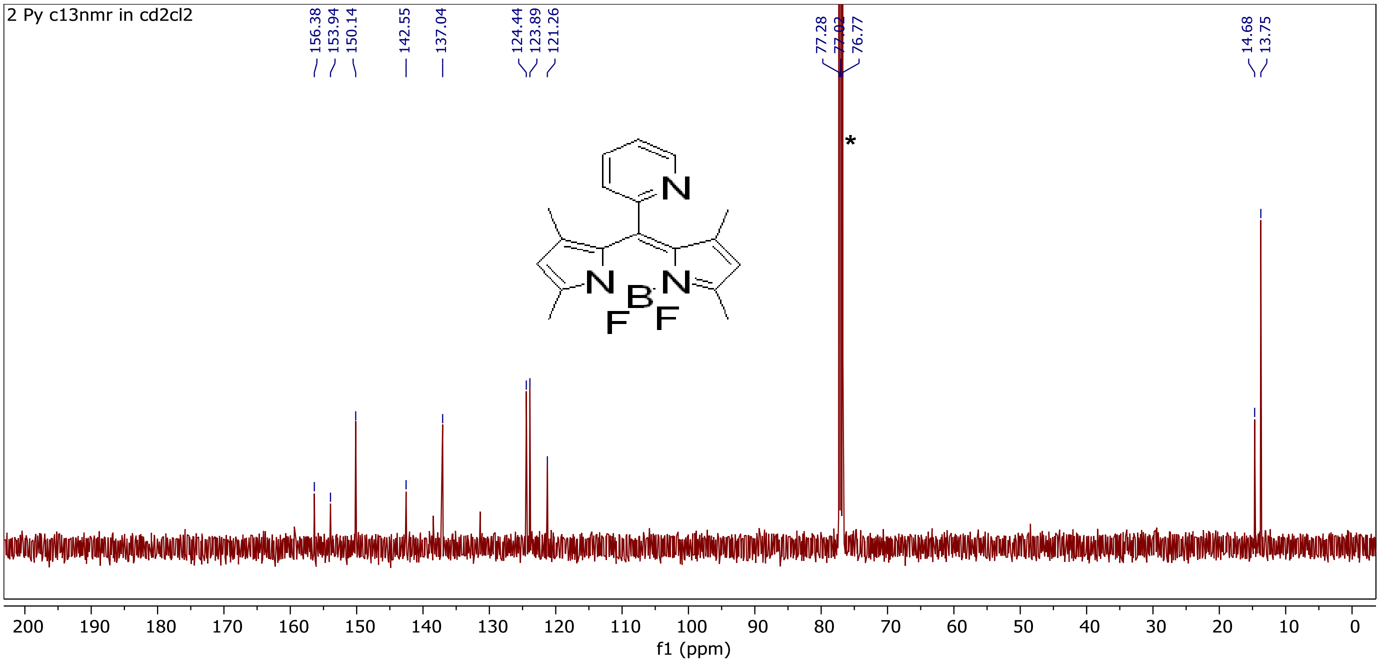

Figure S4. ^{13}C NMR of BODIPY **2Py** in $CDCl_3$

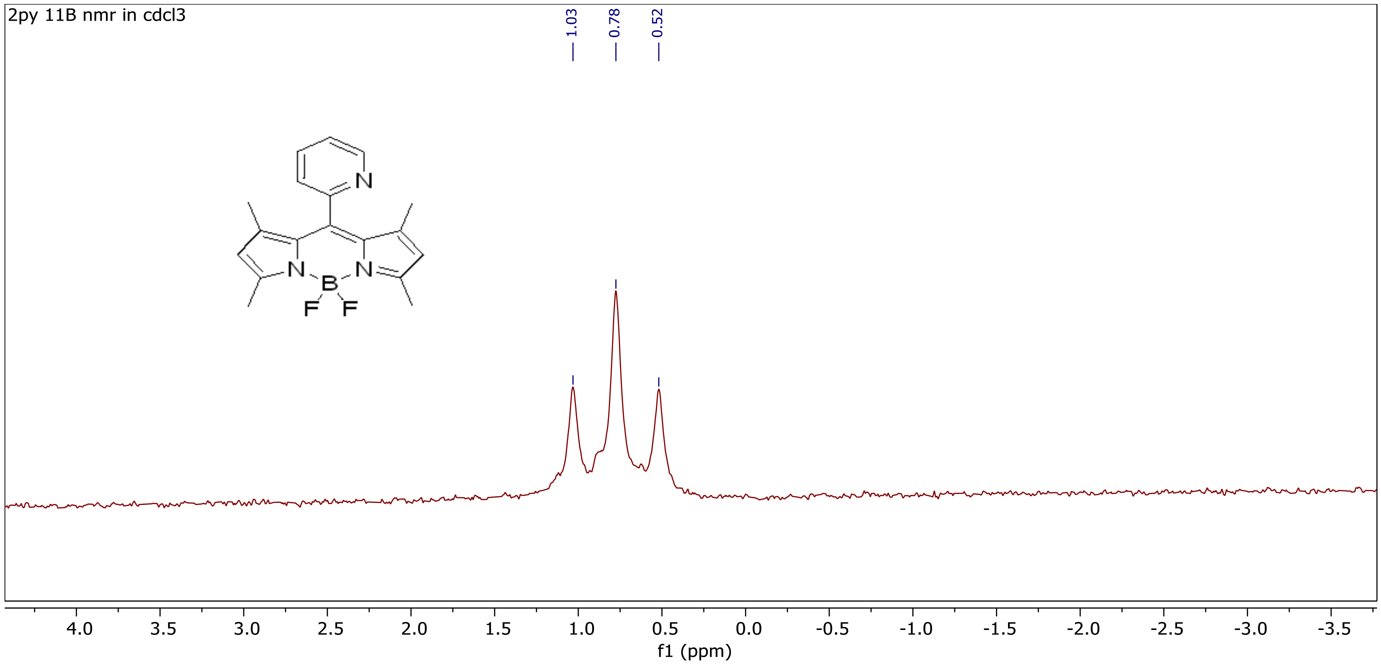

Figure S5. ^{11}B NMR of BODIPY **2Py** in $CDCl_3$

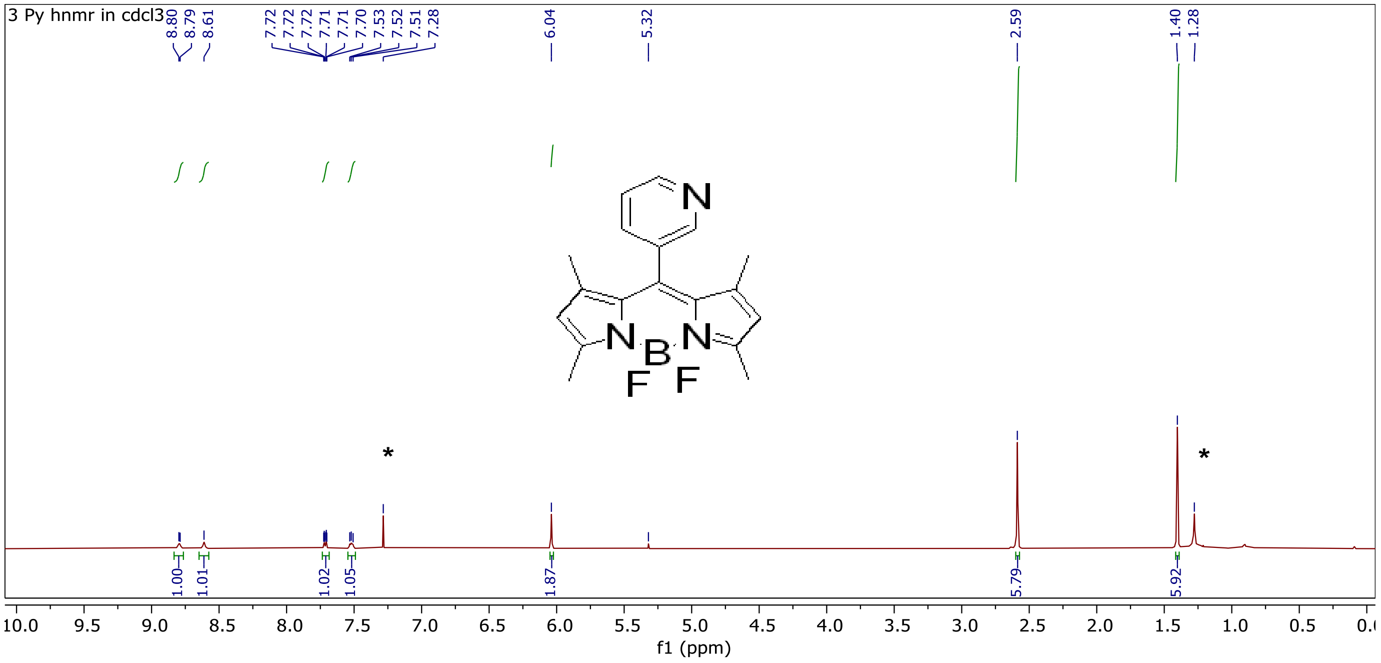

Figure S6. ^{1}H NMR of BODIPY **3Py** in $CDCl_3$

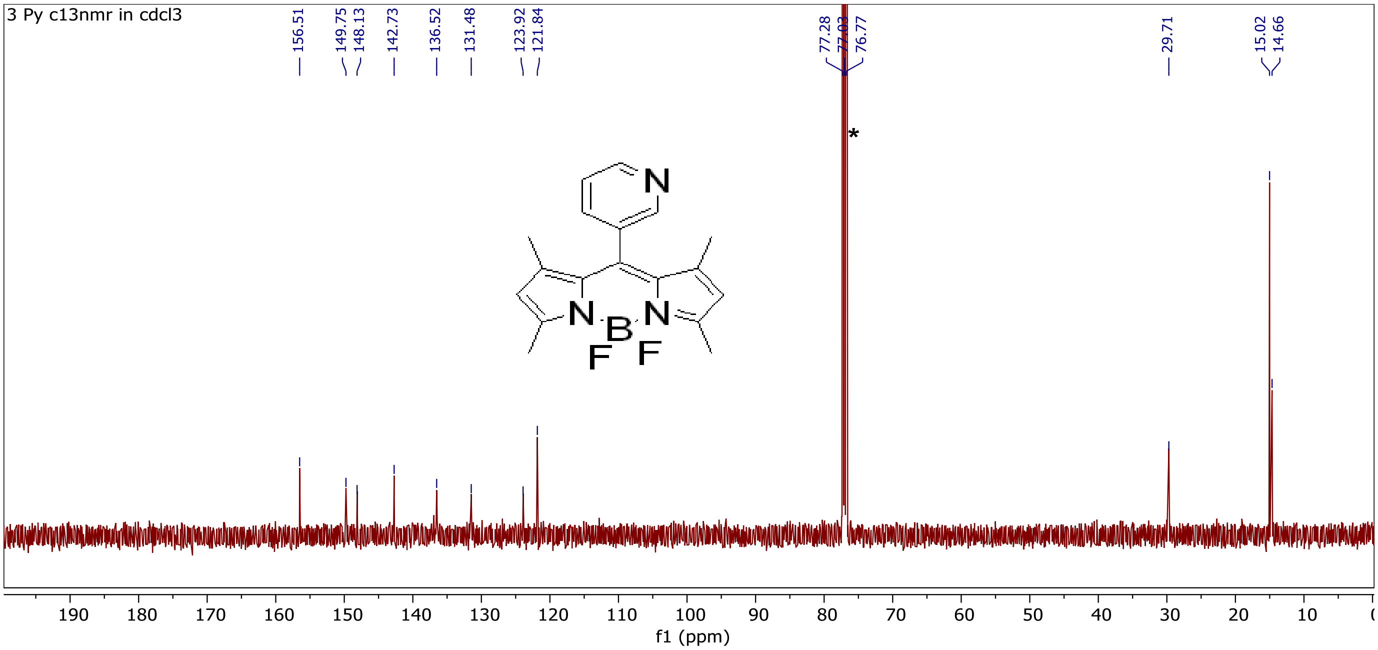

Figure S7. ^{13}C NMR of BODIPY **3Py** in $CDCl_3$

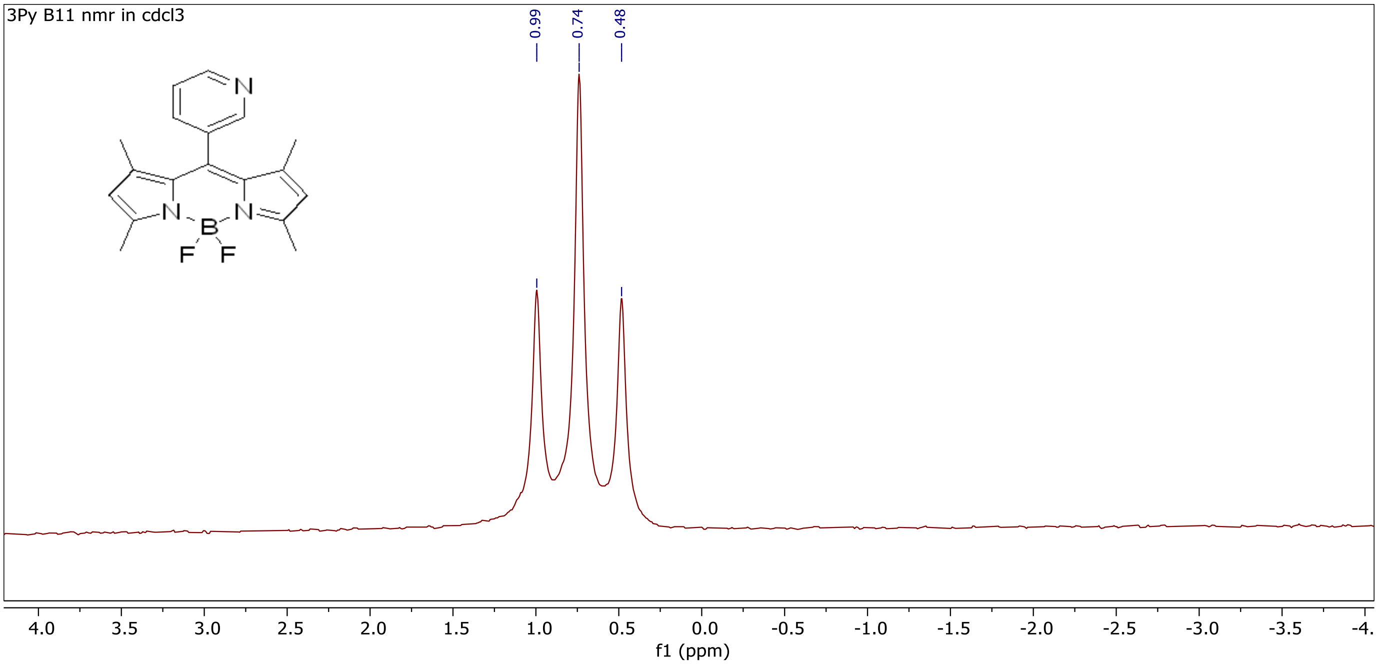

Figure S8. ^{11}B NMR of BODIPY **3Py** in $CDCl_3$

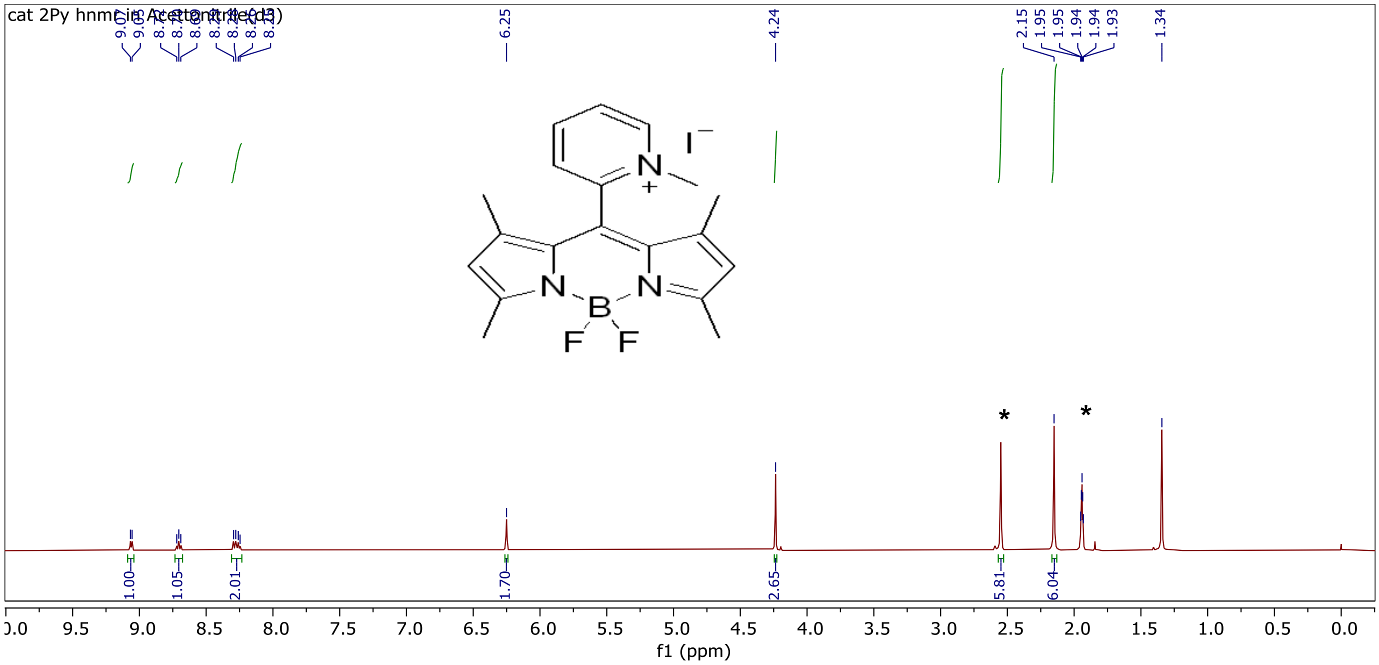

Figure S9. ^{1}H NMR of BODIPY **2catPy** in acetonitrile-d_3

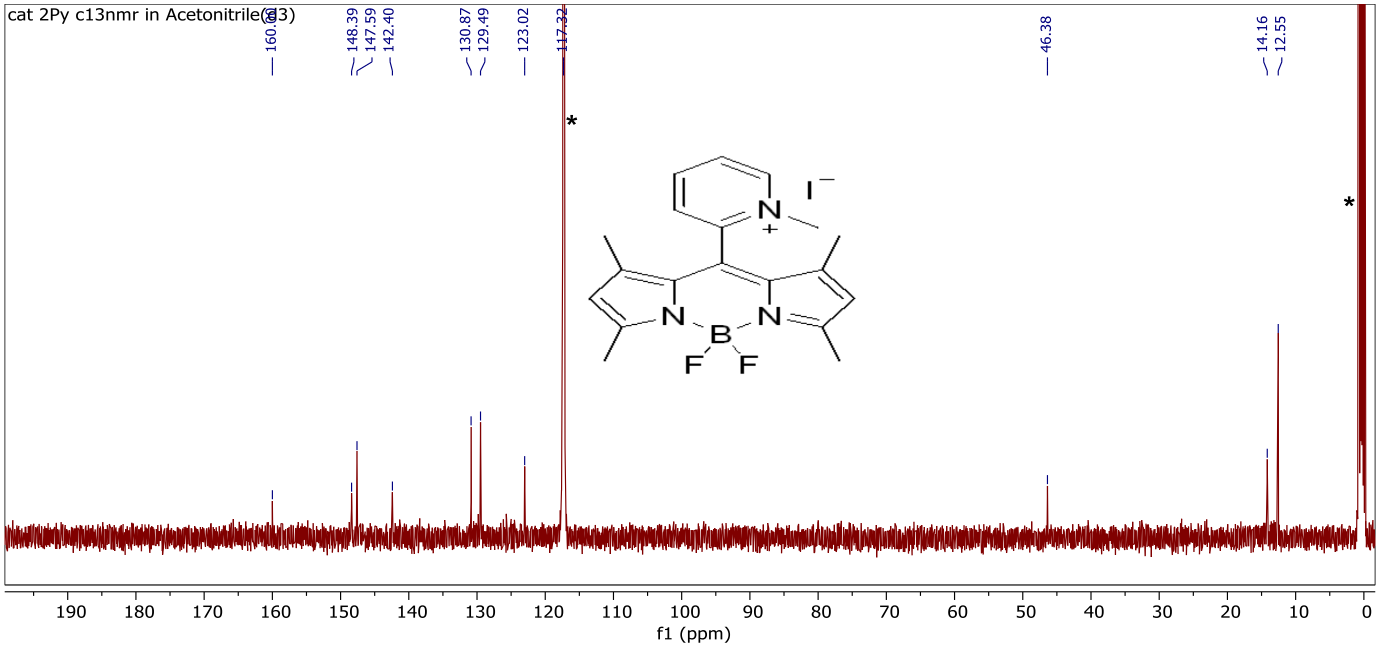

Figure S10. ^{13}C NMR of BODIPY **2catPy** in acetonitrile-d_3

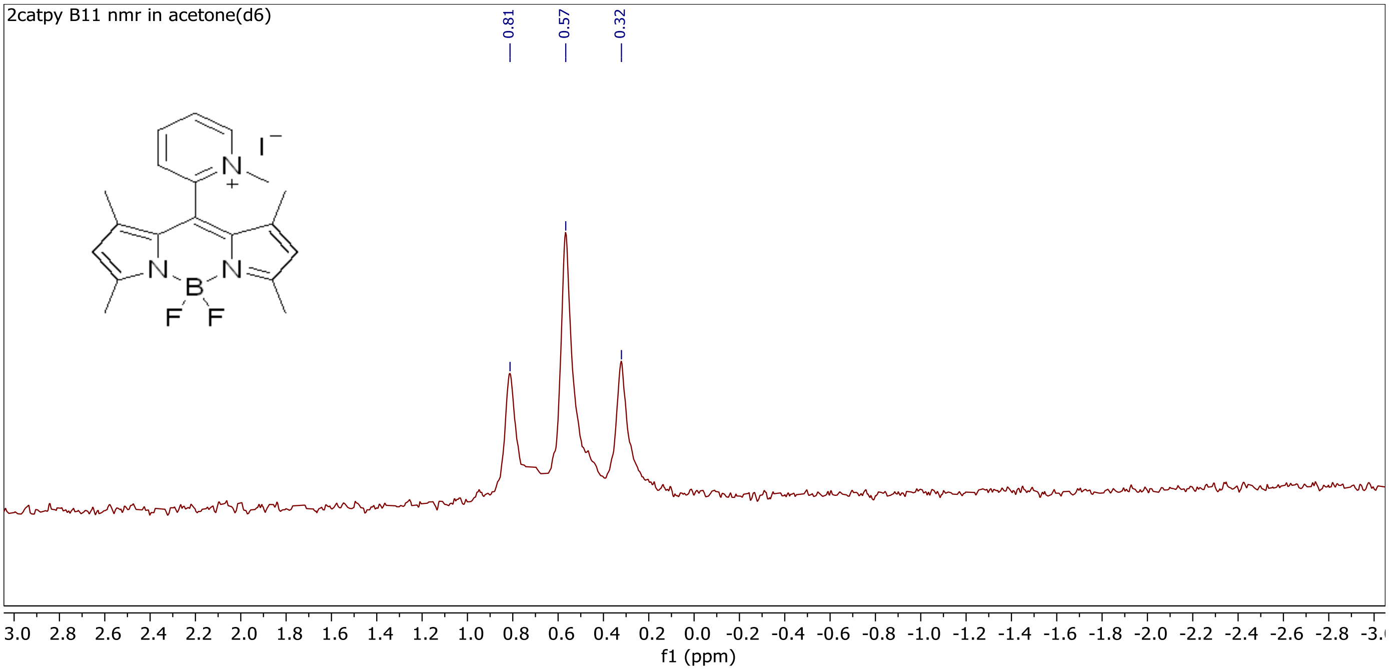

Figure S11. ^{11}B NMR of BODIPY **2catPy** in acetone-d_6

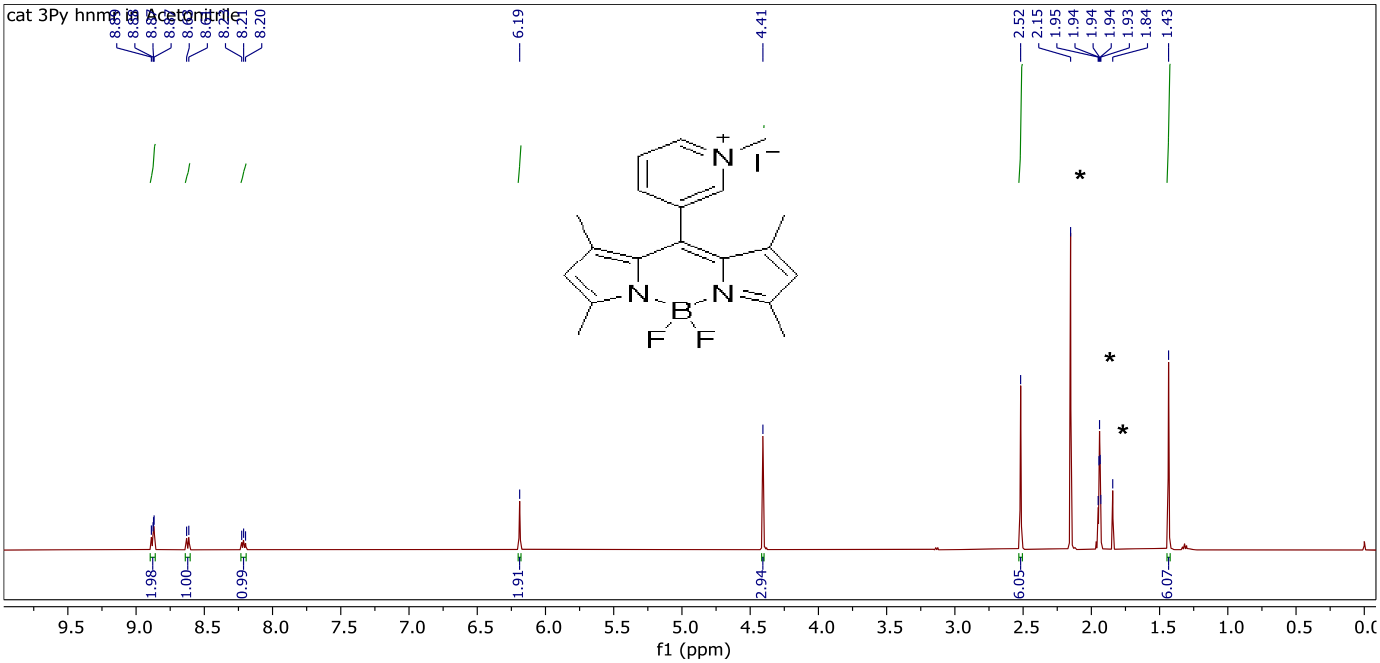

Figure S12. ^{1}H NMR of BODIPY **3catPy** in acetone-d_6

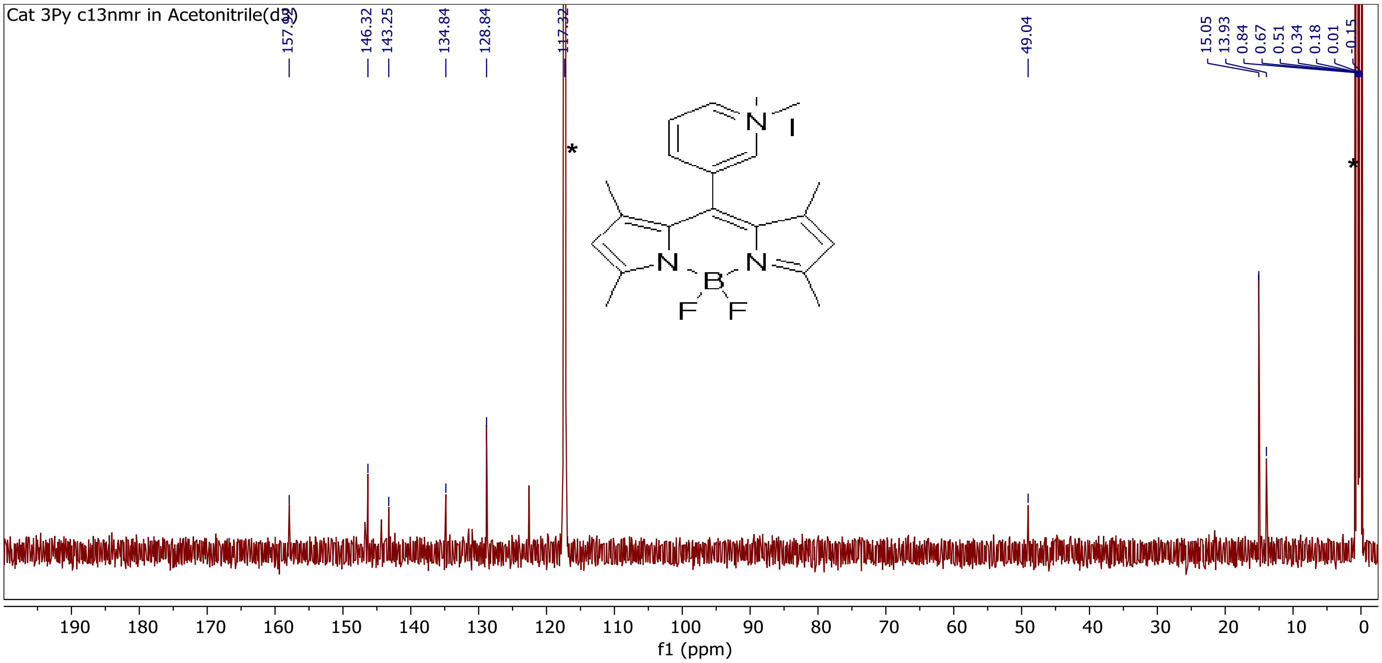

Figure S13. ^{13}C NMR of BODIPY **3catPy** in acetonitrile-d_3

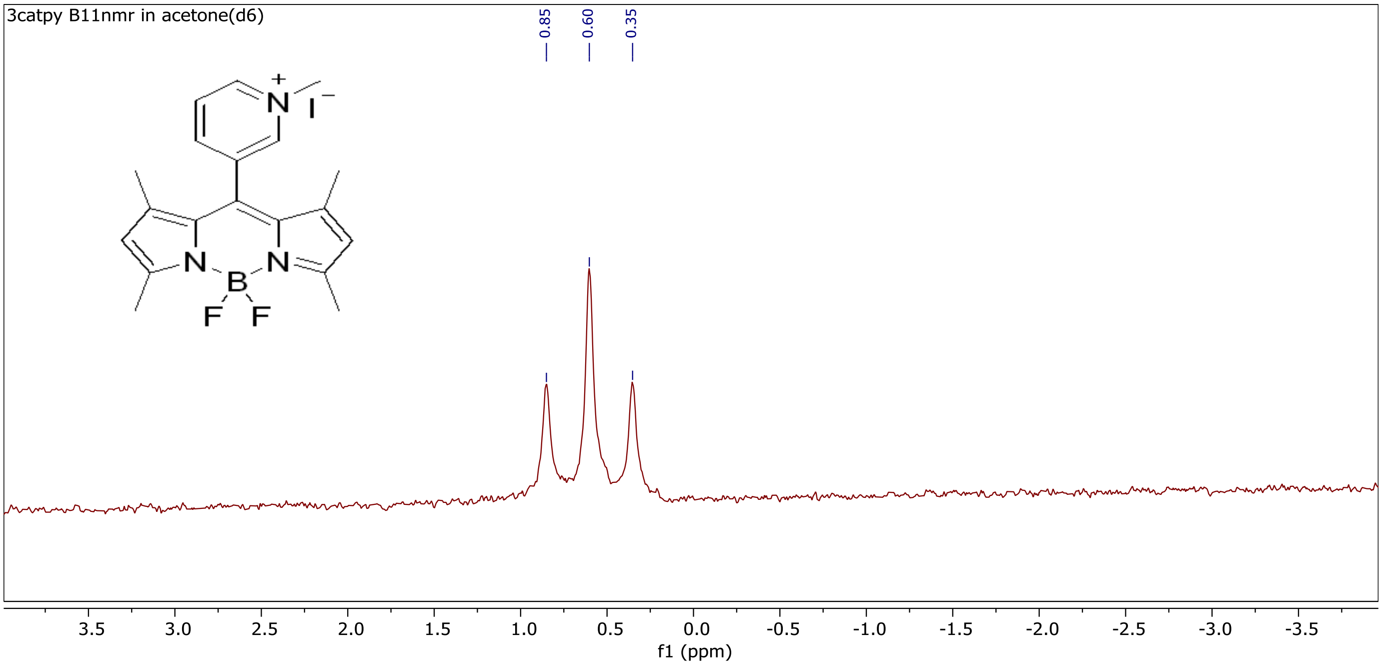

Figure S14. ^{11}B NMR of BODIPY **3catPy** in acetone-d_6

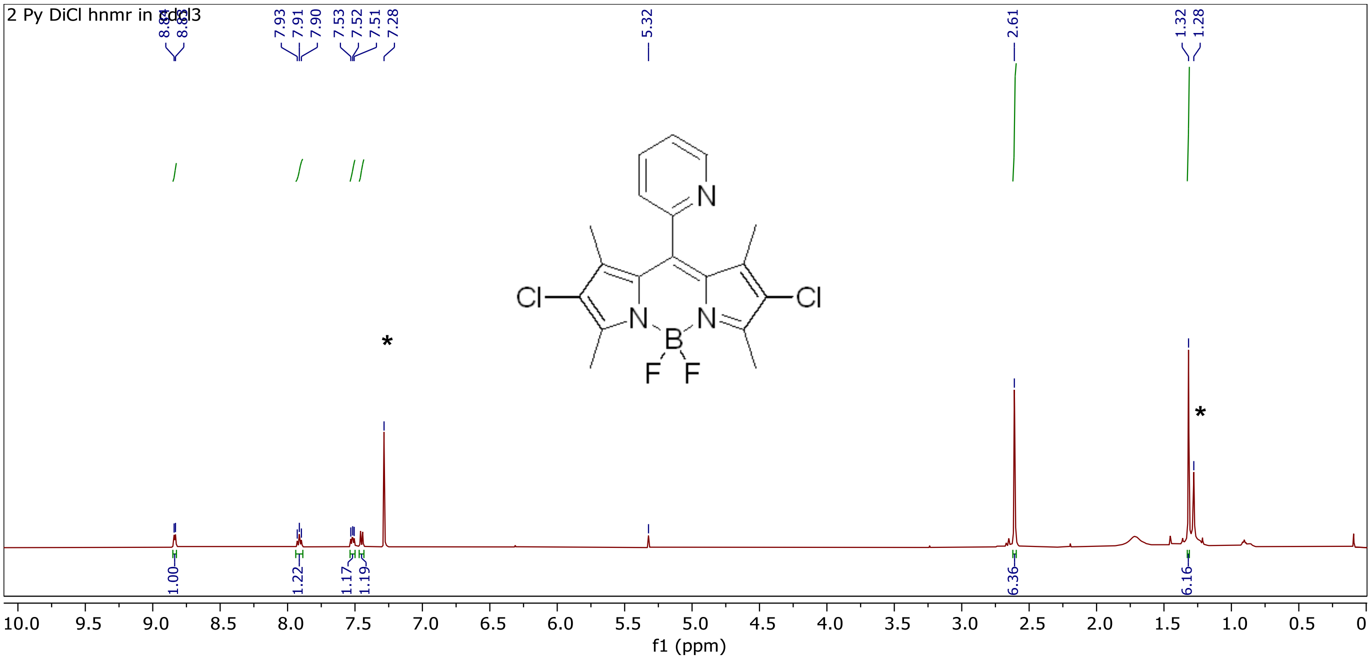

Figure S15. ^{1}H NMR of BODIPY **2PyCl$_2$** in $CDCl_3$

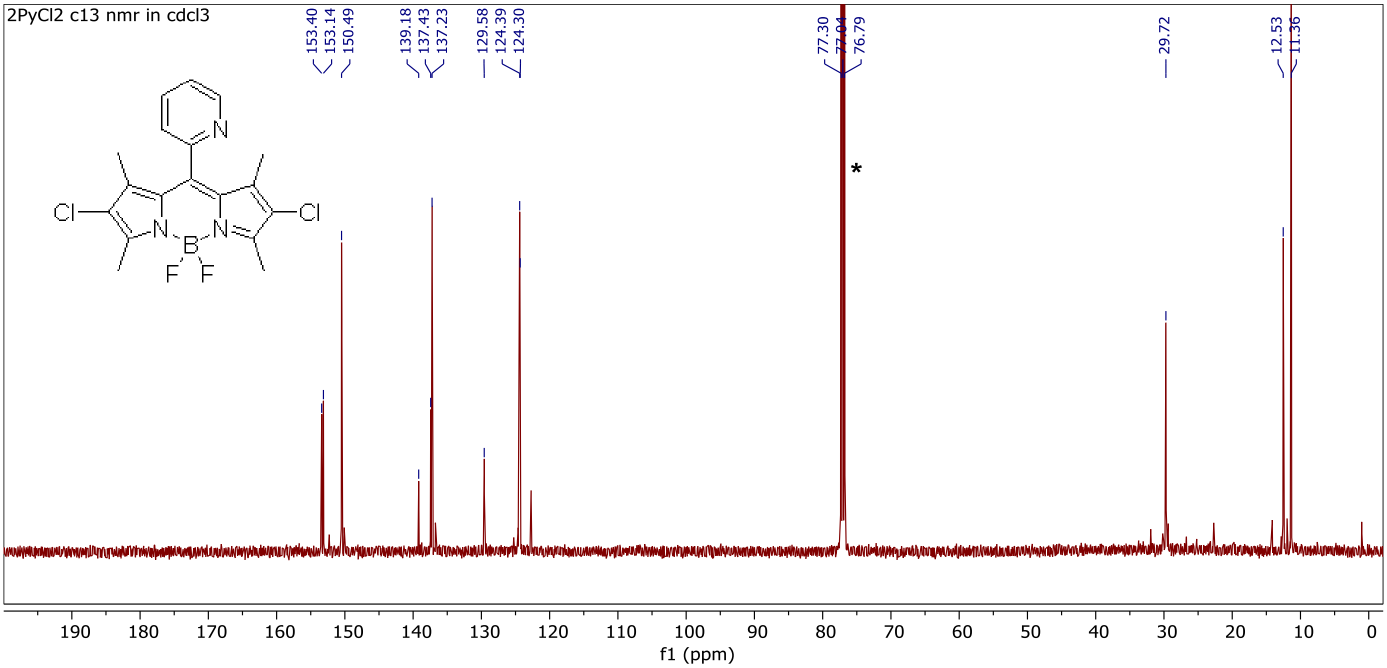

Figure S16. ^{13}C NMR of BODIPY **2PyCl$_2$** in $CDCl_3$

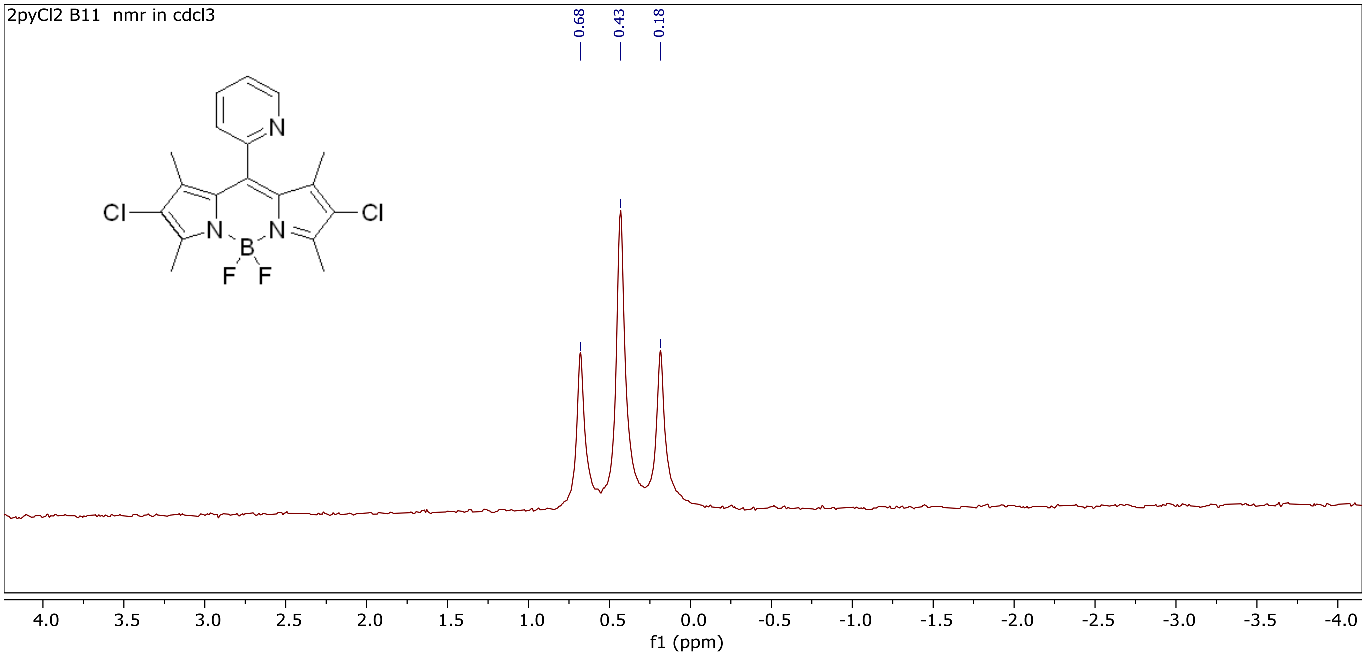

Figure S17. ^{11}B NMR of BODIPY **2PyCl$_2$** in $CDCl_3$

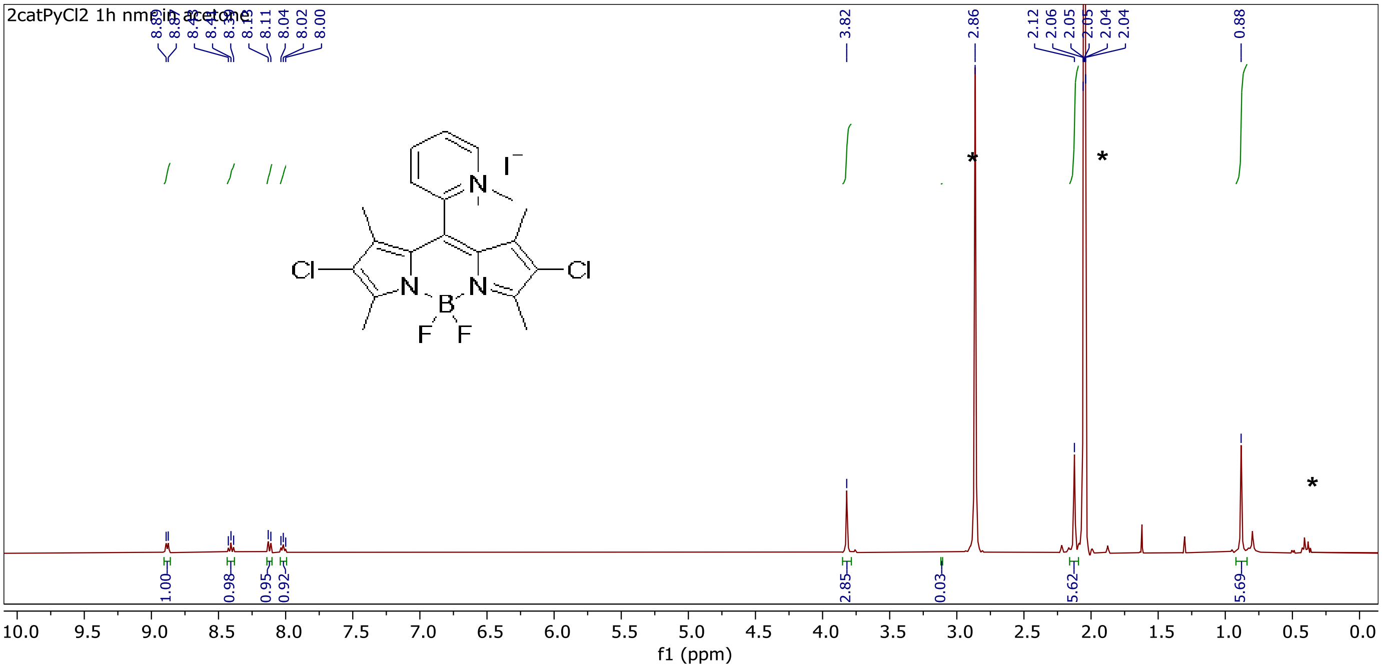

Figure S18. ^{1}H NMR of BODIPY **2catPyCl$_2$** in acetone-d$_6$

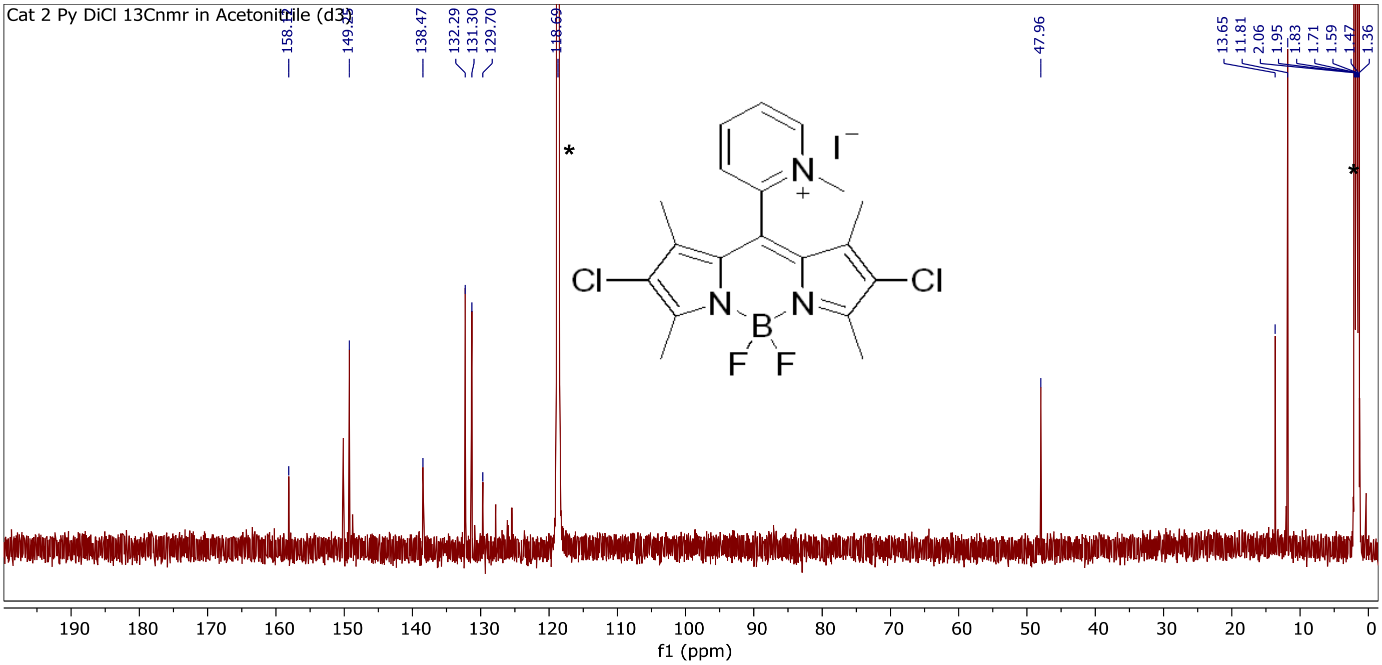

Figure S19. ^{13}C NMR of BODIPY **2catPyCl$_2$** in acetonitrile-d$_3$

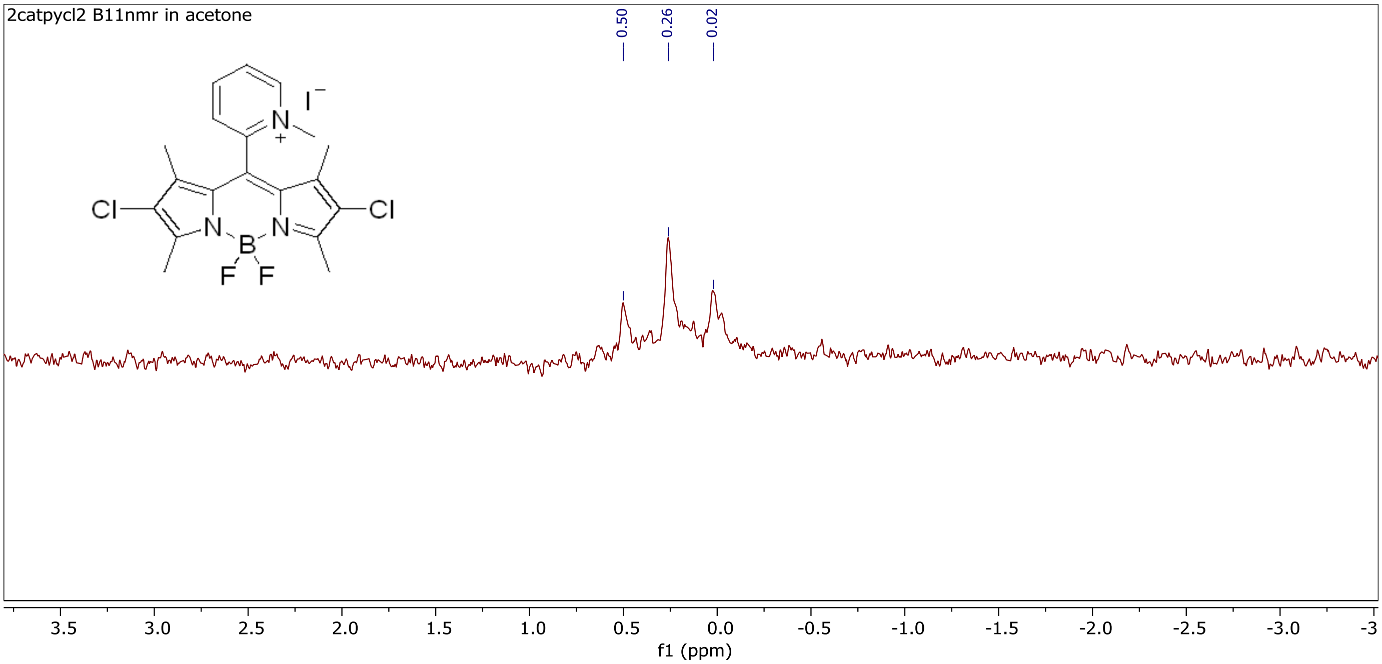

Figure S20. ^{11}B NMR of BODIPY **2catPyCl$_2$** in acetone-d$_6$

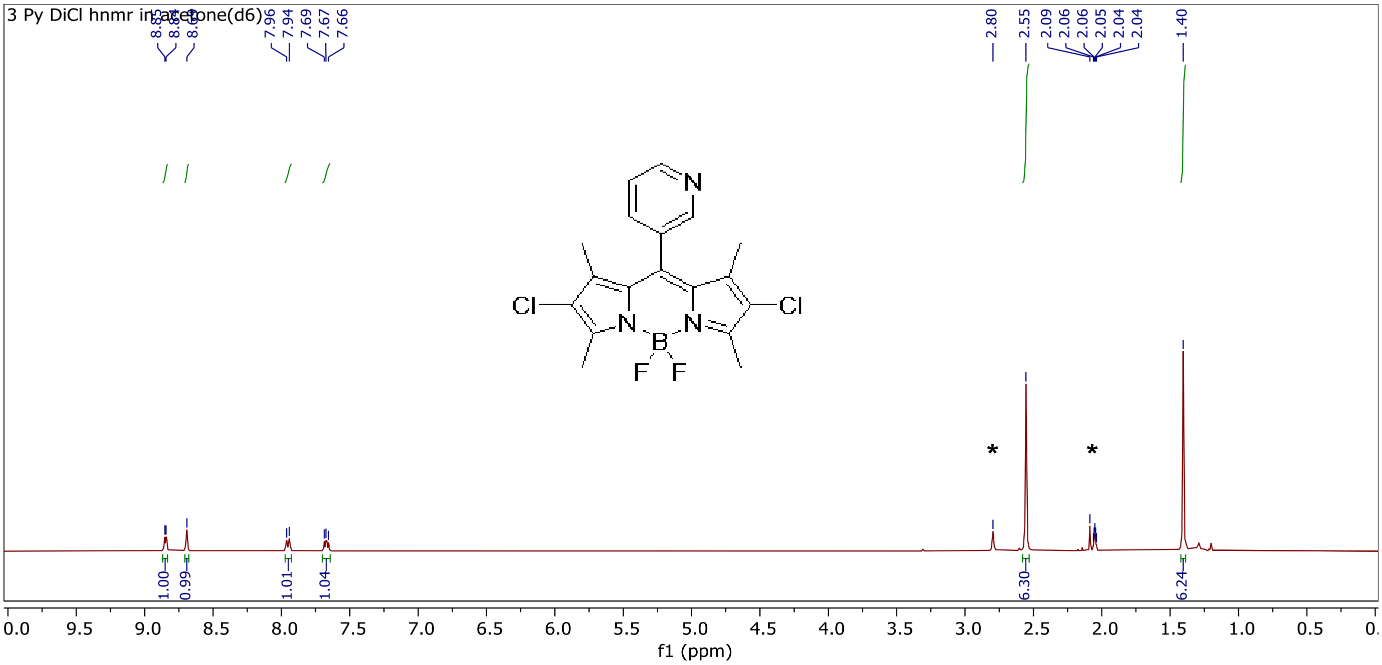

Figure S21. ^{1}H NMR of BODIPY **3PyCl₂** in acetone-d₆

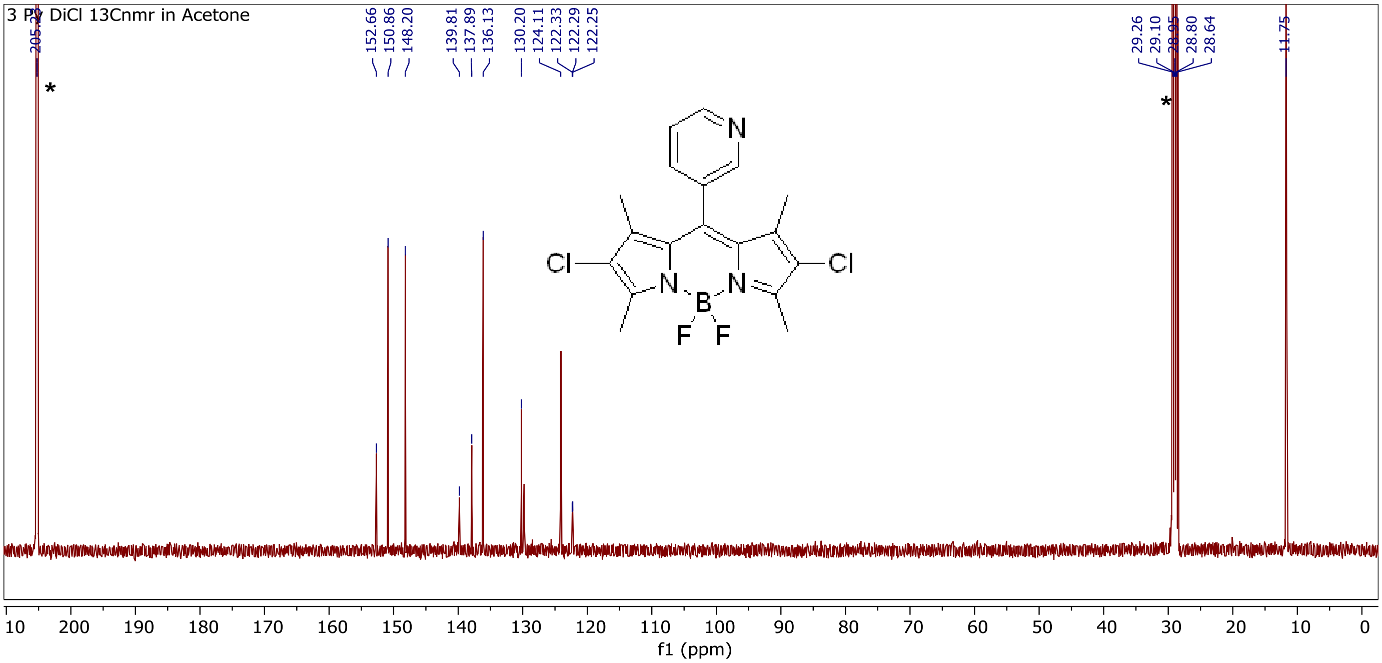

Figure S22. ^{13}C NMR of BODIPY **3PyCl₂** in acetone-d₆

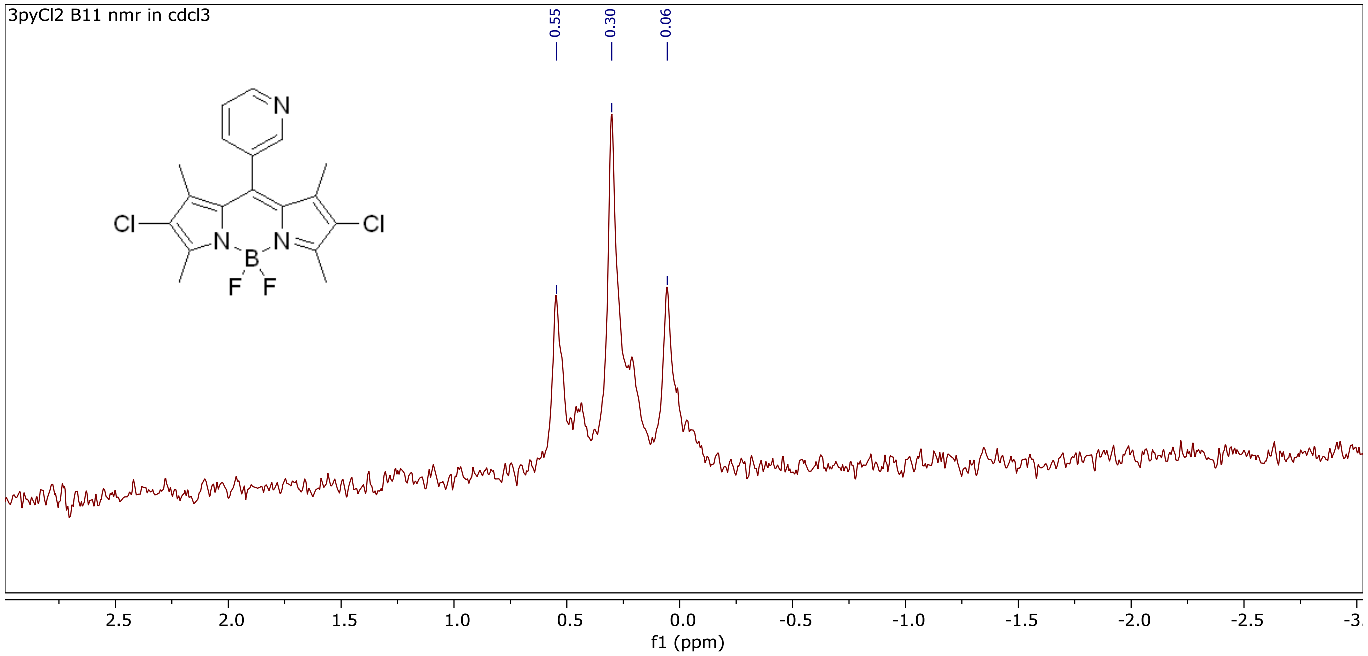

Figure S23. ^{11}B NMR of BODIPY **3PyCl₂** in $CDCl_3$

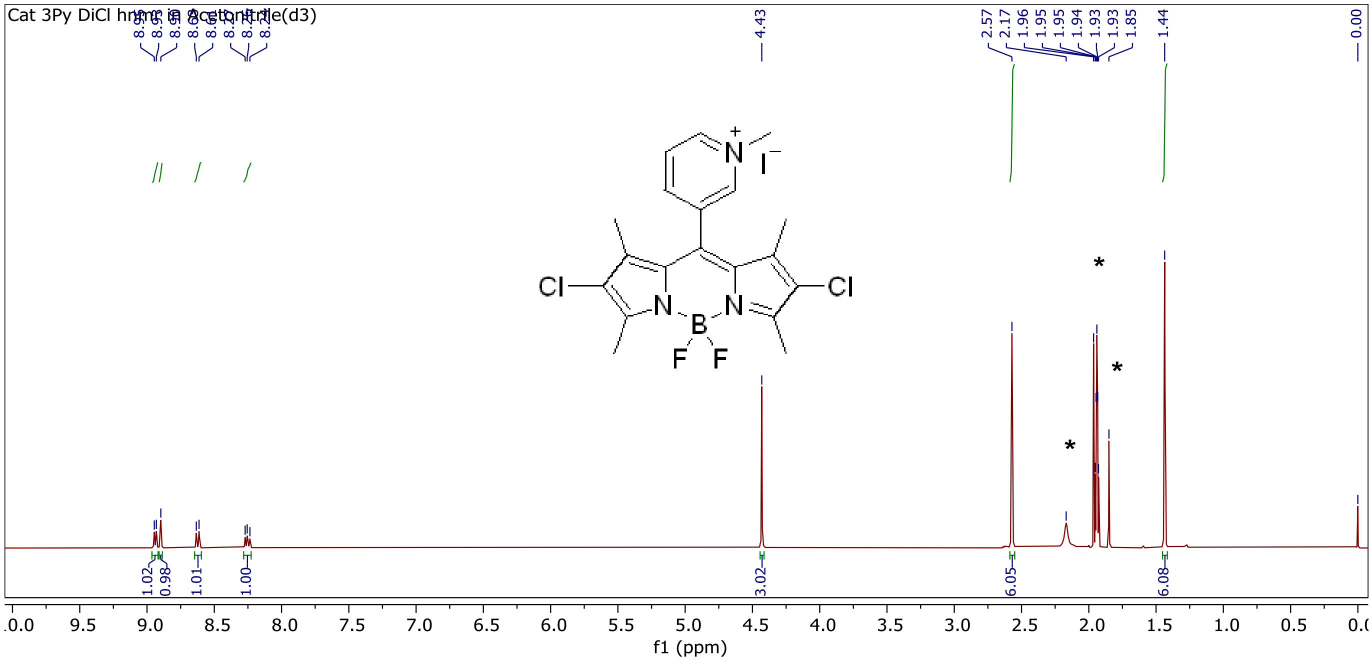

Figure S24. ^{1}H NMR of BODIPY **3catPyCl₂** in acetonitrile-d_3

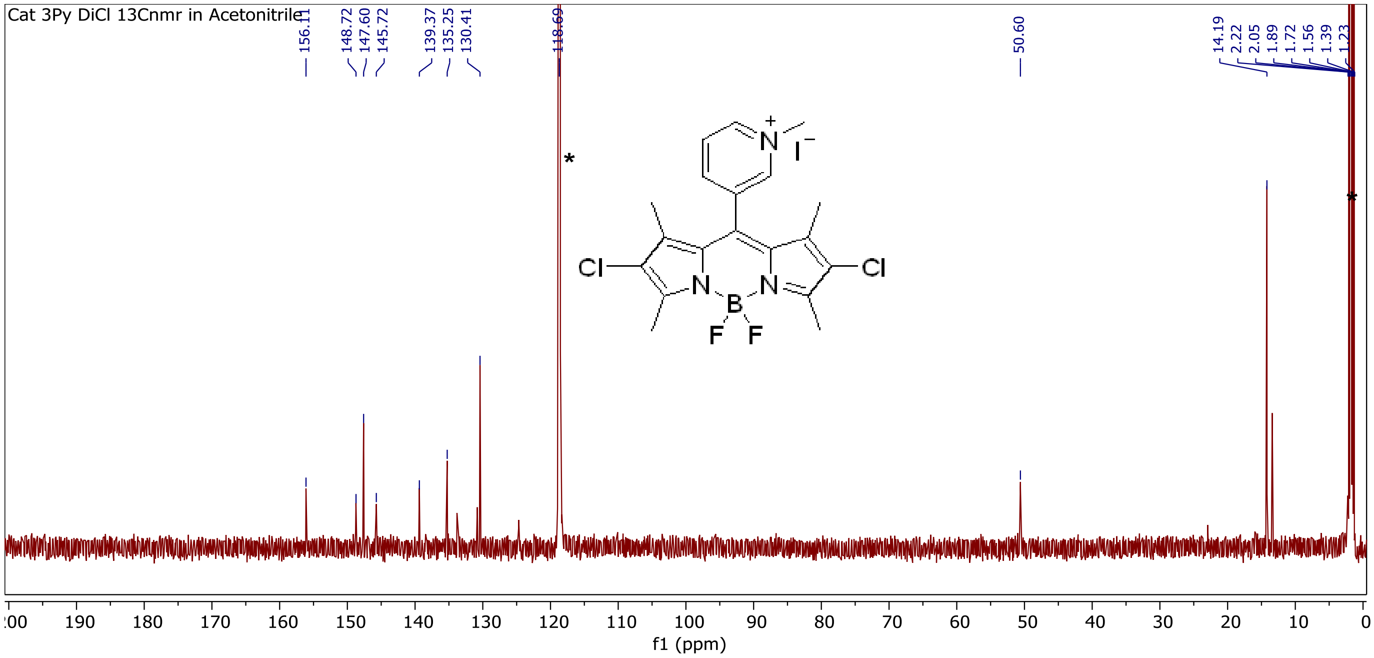

Figure S25. ^{13}C NMR of BODIPY **3catPyCl$_2$** in acetonitrile-d$_3$

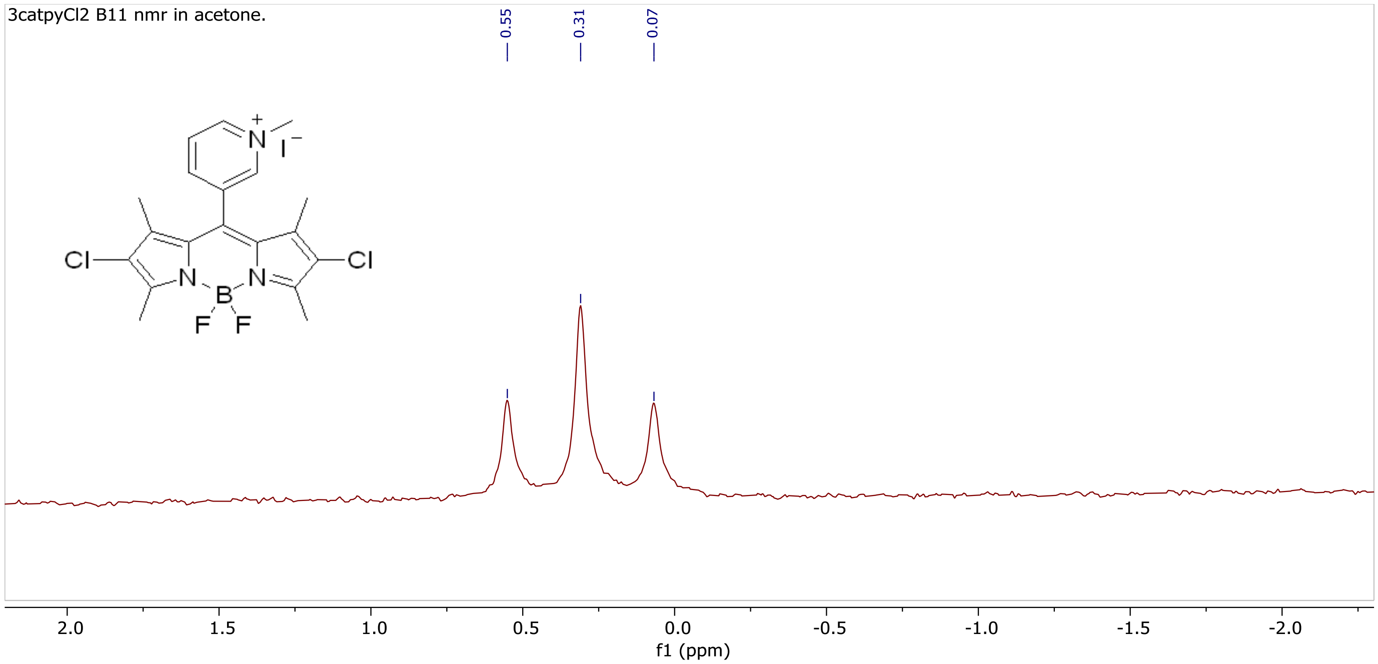

Figure S26. ^{11}B NMR of BODIPY **3catPyCl$_2$** in acetone-d$_6$
